# Feasibility randomised controlled trial to assess the delivery of a novel isometric exercise intervention for people diagnosed with uncomplicated stage 1 hypertension in the National Health Service: key quantitative findings

**DOI:** 10.1136/bmjopen-2024-091219

**Published:** 2025-06-04

**Authors:** Jim D Wiles, Ellie Santer, Melanie Rees-Roberts, Rachel Borthwick, Timothy Doulton, Pauline A Swift, T Pellatt-Higgins, Katie Saxby, Ashley Mills, Katerina Gousia, Douglas MacInnes, Jamie O’Driscoll, Alan West, John Darby, Vanessa Short, Chris K Farmer

**Affiliations:** 1 Sport, Exercise and Rehabilitation Sciences, Canterbury Christ Church University, Canterbury, UK; 2 Centre for Health Services Studies, University of Kent, Canterbury, UK; 3 Department of Renal Medicine, East Kent Hospitals University NHS Foundation Trust, Canterbury, UK; 4 Renal Services, Epsom and Saint Helier University Hospitals NHS Trust, Carshalton, UK; 5 Faculty of Health and Wellbeing, Canterbury Christ Church University, Canterbury, UK; 6 Public Co-applicant, Canterbury, UK; 7 Newton Place Surgery, Faversham, UK

**Keywords:** Hypertension, Clinical Trial, Exercise

## Abstract

**Objectives:**

The aim of this study was to determine the feasibility of delivering personalised isometric exercise (IE) for people with stage 1 hypertension. Is it feasible to deliver an isometric wall squat intervention in the National Health Service and what sample size is required to conduct an appropriately powered effectiveness randomised controlled trial (RCT)?

**Design:**

Randomised controlled open-label multicentre feasibility study of IE compared with standard care in unmedicated people with stage 1 hypertension.

**Setting:**

Initially, the study aimed to recruit through primary care, but this process coincided with the advent of the COVID-19 pandemic. Therefore, we shifted focus to direct-to-public advertising and delivery in secondary care.

**Participants:**

People with unmedicated stage 1 hypertension aged over 18 able to perform IE were included. Patients were excluded if average home systolic blood pressure (sBP) <135 mm Hg; were unable to undertake the study intervention; had a previous history of diabetes mellitus, ischaemic heart disease, moderate-severe valvular heart disease, arrhythmia, stroke or transient ischaemic attack, aortic aneurysm, peripheral arterial disease and uncorrected congenital heart condition; stage 3b chronic kidney disease or worse; heart failure; enrolled in another clinical trial; pregnant or breastfeeding. 41 participants (57±15 years), 59% women, were randomised.

Intervention participants were randomised (1:1) to either standard lifestyle advice or an individualised isometric wall squat prescription, performed 4×2-min bouts three times a week for 6 months.

**Primary and secondary outcome measures:**

We assessed deliverability, attrition, adherence and variance in blood pressure (BP) change.

**Results:**

IE was found to be easily deliverable to all participants. At 6 months, 34% had withdrawn. Of those who completed IE, 85% of their sessions were at the correct intensity, meeting our retention criterion for success. Variance in BP change was 14.4 mm Hg. The study was not powered to show a difference in BP between groups; however, BP reductions were seen in the intervention group at all study time points compared with baseline. There were no adverse events related to study participation.

**Conclusions:**

We met our a priori recruitment criteria which allowed us to calculate a sample size (n=542) for a full RCT. The results demonstrate good acceptability and adherence rates to the treatment protocol. Our results show a signal towards a consistent sBP reduction in the IE group compared with baseline.

**Trial registration number:**

NCT04936022 (https://classic.clinicaltrials.gov/ct2/show/NCT04936022?cond=isometric+exercise&draw=2&rank=7); registry identifier: ISRCTN 13472393.

**STRENGTHS AND LIMITATIONS OF THIS STUDY**

- First study delivering an isometric exercise intervention in the National Health Service.
- Established a remote supervised blood pressure measurement in the home.
- Recruitment challenges in the COVID-19 pandemic.
- Lack of diversity and limited geographical location.

## Introduction

 The global age-standardised prevalence of hypertension in women and men (30–79 years) is 32% and 34%, respectively, which disproportionately affects lower socioeconomic and underserved demographics.^1^ Systolic blood pressure (sBP) increases linearly with age after 30 years in industrialised populations^2^ and is the leading level 2 risk factor for attributable deaths, accounting for around 10.8 million global deaths annually.^3^ A major concern is that only 30% of patients with high BP are treated effectively.^4^ Of those on anti-hypertensive therapy, up to 50% of people fail to achieve their target, mainly due to non-adherence (estimated 30%–50%),^5 6^ with side effects of medication often cited.^7 8^ The estimated direct cost associated with hypertension in the UK is £5 billion.^9^ With an ageing population, the prevalence of hypertension will also increase.

The importance of lifestyle changes for patients with hypertension should not be overlooked.^10 11^ Exercise lowers blood pressure (BP)^12^,^14^ and may be as effective as medication in controlling BP.^15^ However, low adoption and high attrition rates are common.^16 17^ Around 1:3 women and 1:4 men do not do enough physical activity to stay healthy.^18^ Guidance in relation to exercise is generic with the same recommendations for those with and without hypertension.^18^ Data also suggest that people with hypertension are less physically active than those without.^19^ Additionally, a recent study showed only one in five general practitioners (GPs) are broadly or very familiar with physical activity guidelines, and 72% of GPs do not convey the benefits of physical activity.^20^ To promote lifestyle exercise changes, people need easy, effective and manageable interventions as a first-line option for managing their BP. Current physical activity guidelines prioritise at least 150 min of moderate intensity aerobic activity a week. Frequently cited barriers to exercise are lack of time and resources,^21^ which may help to explain the low adoption (<8%), poor adherence (67%) and high attrition rates (50%) to this relatively large amount of aerobic exercise often reported.^16^

Isometric exercise (IE) training has been consistently shown to reduce BP in both sexes.^22^ Only 24 min of IE weekly is required to achieve reductions in BP of 12/6 mm Hg in unmedicated hypertensives, which can be performed at home without costly equipment.^23^ Moreover, a meta-analysis has demonstrated that IE is the most effective exercise mode to reduce sBP.^24^ The effectiveness of IE has never been tested within a national healthcare setting, nor confirmed in any UK community-based randomised controlled trials (RCTs). As such, this feasibility study aimed to inform the design of a large-scale RCT to evaluate the efficacy and mechanisms of wall squat IE to lower BP in UK National Health Service (NHS) patients who have sBP between 140 and 159 mm Hg not taking anti-hypertensive treatment.^25^

This paper reports the quantitative results of a feasibility study^25^ focusing on whether a RCT of IE in uncomplicated hypertension was an appropriate trial design and was feasible regarding (i) recruitment and retention and (ii) adherence to protocol. In addition, we needed to estimate the full trial sample size following the determination of the variance in BP change. It is important to recognise that this paper did not set out to cover all the aims and objectives outlined in the original protocol paper.^25^ Qualitative results are discussed in Rees-Roberts *et al*,^26^ and recruitment issues in primary care during the COVID-19 pandemic are critically appraised in Farmer *et al*.^27^

## Objectives

### Aim

To determine the feasibility of delivering personalised IE for people with hypertension: quantitative results.

### Research questions

Is an isometric wall squat intervention feasible to deliver in the NHS and what sample size is required to conduct an appropriately powered effectiveness RCT?

### Primary outcome measures

- To determine the variance in BP change and to enable a sample size calculation for a RCT.

### Secondary outcome measures

- Evidence the fidelity of completion of IE.
- Determine 4-week, 3-month and 6-month adherence rates.
- Determine recruitment and attrition rates.
- Determine delivery cost.

## Methods

### Study design

This study was a multicentre feasibility RCT of IE for patients with sBP 140–159 mm Hg not taking anti-hypertensive treatment, carried out in south-east England.

All procedures conformed to the Declaration of Helsinki, and the National Research Ethics Committee approved the study (REC ref: 20/LO/0422, IRAS ID: 274676). All participants provided written informed consent.

### Patient and public involvement

Study design and delivery has benefited from lay members of the project management group. Their previous experience and insight have been invaluable when commenting on important issues, offering a patient perspective to all elements of the design including patient access to technology, optimising reminder texts (to mitigate attrition) and improving the participant documents and resources. Patient and public involvement and engagement was essential in successfully adapting the study for remote delivery during the pandemic and beyond.

### Setting

Initially, the study aimed to recruit through primary care, but this process coincided with the advent of the COVID-19 pandemic. Therefore, as discussed in Farmer *et al*,^27^ we shifted focus to direct-to-public advertising.

### Participants

**Figure 1:**
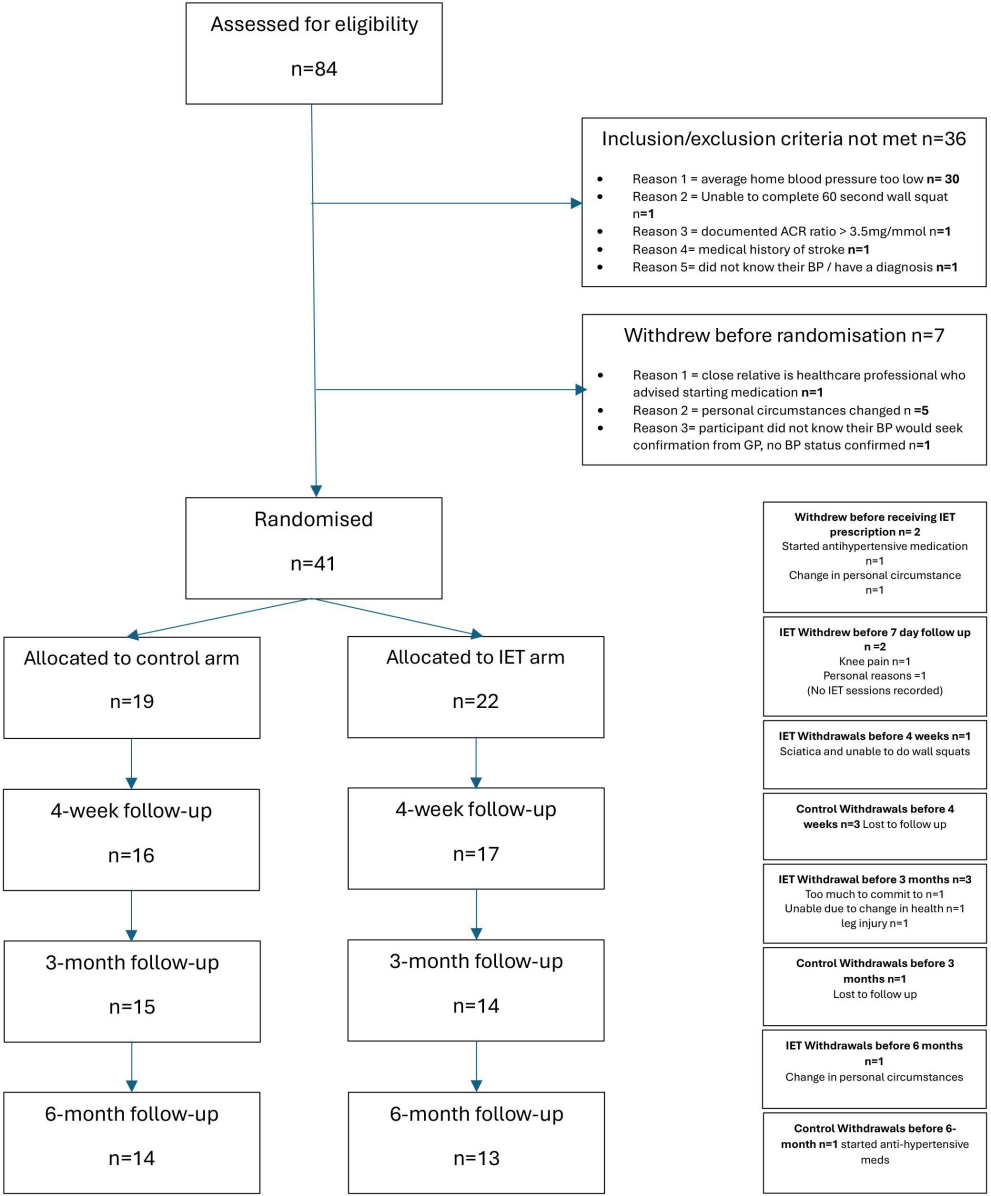
Consolidated Standards of Reporting Trials (CONSORT) flow diagram illustrating recruitment and follow-up. ACR, albumin to creatinine ratio; BP, blood pressure; GP, general practitioner; IET, isometric exercise training.

Overall, 84 unmedicated participants with stage 1 hypertension consented to enter the study, seven withdrew prior to screening and 36 were screen failures (figure 1). In total, 17 male and 24 female patients (56.6±14.6 years) with a screening sBP between 140 and 159 mm Hg were recruited (table 1). The first participant was recruited on 26 March 2021, the last on 30 November 2022 and the last follow-up date was 21 June 2023.

**Table 1:** Demographic profile of participants

	Control (n=19)	Isometric exercise (n=22)
Male n (%)	8 (42.1)	9 (40.9)
Age (years), mean (SD)	56.2 (14.3)	57.0 (15.2)
Age≥50 years, n (%)	14 (73.7)	16 (72.7)
Height (cm), mean (SD)	170.3 (12.1)	171.0 (9.9)
Weight (kg), mean (SD)	80.7 (15.5)	79.1 (15.1)
Systolic BP (mm Hg) mean (SD)	153.7 (13.2)	152.3 (12.3)
Diastolic BP (mm Hg) mean (SD)	89.7 (7.3)	94.0 (8.8)
Smoking status n (%)		
Smoker	1 (5.3)	2 (9.1)
Previous smoker	8 (42.1)	6 (27.3)
Non-smoker	10 (52.6)	14 (63.6)

Data presented as mean and SD or % where indicated.

BP, blood pressure.

Patients were excluded if they were taking anti-hypertensive medication; had white coat hypertension (as evidenced by averaged home sBP <135 mm Hg); were unable to undertake the study intervention (IE); had a previous history of diabetes mellitus (type 1 or type 2), known or suspected ischaemic heart disease (including myocardial infarction and/or angina and/or coronary revascularisation procedure), moderate or severe stenotic or regurgitant heart valve disease, atrial or ventricular arrhythmia, stroke or transient ischaemic attack, aortic aneurysm and/or peripheral arterial disease, uncorrected congenital or inherited heart condition; had an estimated glomerular filtration rate <45 mL/min (calculated using chronic kidney disease epidemiology calibration or modification of diet in renal disease formulae and taking most recent documented results); had a documented left ventricular ejection fraction <45% and/or left ventricular hypertrophy (by either echocardiography or standard ECG criteria, eg, Sokolow-Lyon); had a documented urine albumin to creatinine ratio >3.5 mg/mmol; were unable to provide informed consent; were enrolled in another Clinical Trial of an Interventional Medicinal Product or Medical Device or other interventional study; and, if female, were pregnant or currently breastfeeding. Then, finally, if there is any medical condition that, in the opinion of the investigator, would make the participant unsuitable for the study.

Participants were recruited in Kent and Surrey from hospital hypertension clinics and GP surgeries. The majority of participants were recruited through advertising (including posters and social media).

**Figure 2:**
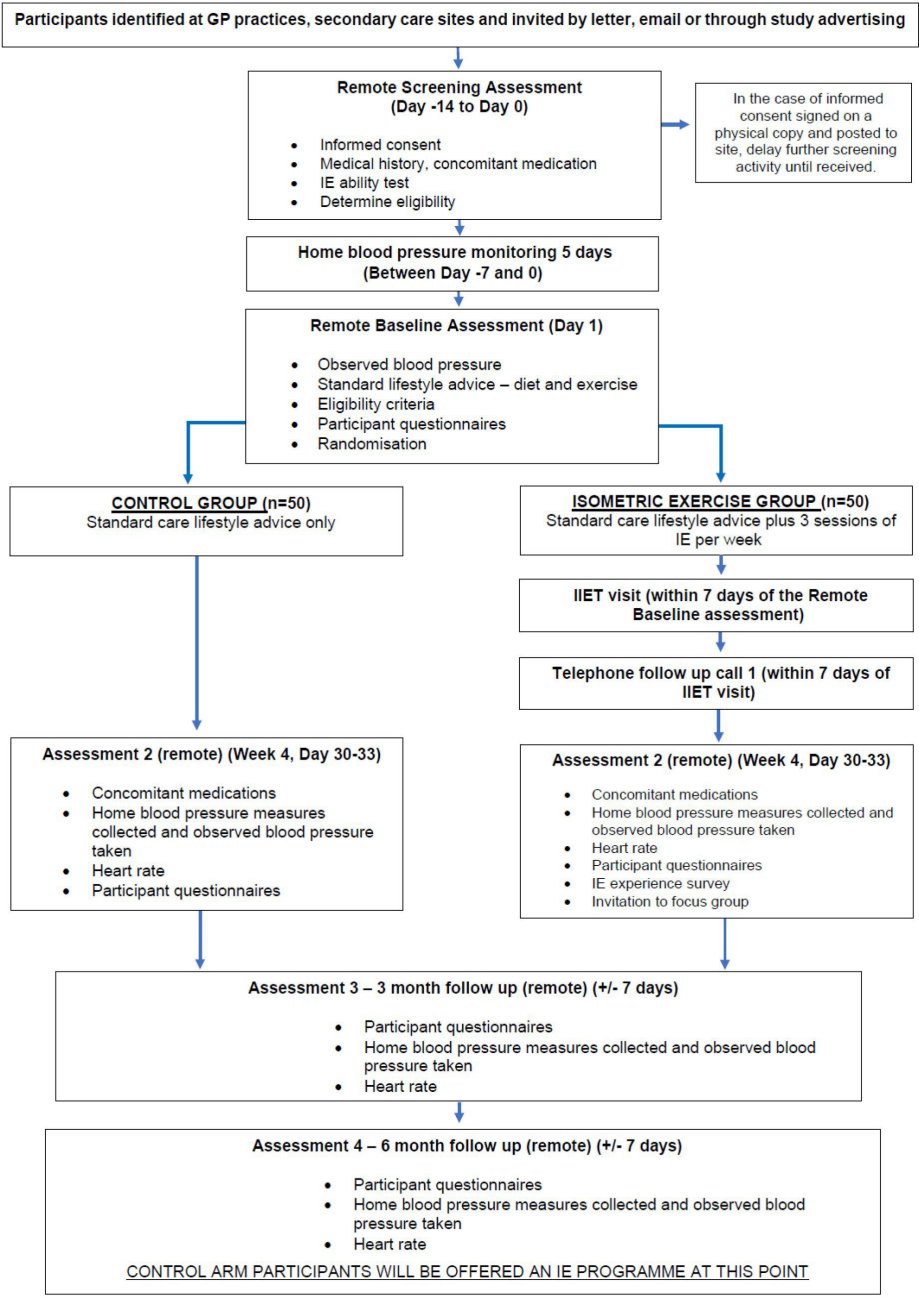
Trial flow chart. GP, general practitioner; IE, isometric exercise; IIET, incremental isometric exercise test.

Once participants had been identified, a remote screening assessment was performed where informed consent was given. The control group agreed to four further remote appointments (video call from home) and one telephone call. Those in the IE group had one in-person visit in a local clinic setting followed by three remote appointments. In addition to supervised BP measurements, participants completed questionnaires on diet, exercise and quality of life (QOL) at each visit. At the end of the follow-up period, all control participants were offered an IE training programme (figure 2).

### Intervention

Following the initial screening visit, eligible patients were sent a home BP monitor (Omron M3 Intellisense, Omron Electronics Ltd., UK) and instructions on use following British and Irish hypertension guidelines (http://bihsoc.org/wp-content/uploads/2017/11/BP-Measurement-Poster-Automated-2017.pdf). They took three readings to confirm BP status. Eligible patients were randomised in a 1:1 ratio (using a commercial randomisation service) to receive either IE or control for 6 months. At each study visit, BP was recorded using video-supervised home BP readings. This involved a trained research nurse carrying out a virtual consultation in real time using video and sound. Participants were witnessed taking their BP according to the study protocol, and the BP readings were confirmed visually on the video call.

Lifestyle advice was provided to all participants based on NHS guidelines (https://www.nhs.uk/conditions/high-blood-pressure-hypertension/treatment/). For those allocated to the IE group, an additional in-person visit took place within 7 days of the remote baseline assessment. During this visit, the participants carried out an incremental isometric exercise test (IIET), the results of which were used to prescribe the correct wall squat intensity for future IE. This was determined by the knee joint angle required to elicit a target heart rate (HR) of 95% HR_peak_. Target heart rate range (THRR) was established for each participant to ensure that future IE training sessions were of the required intensity.^28^ At the end of this session, the participant was taken through the necessary information to successfully complete subsequent wall squat training at home (for full protocol, see Wiles *et al*^25^). Each IE training session comprised four bouts of 2-min wall squats with 2-min recovery in between, with HR recorded at the end of each bout. Participants were asked to perform three IE training sessions a week, ideally on alternate days to allow for adequate between-session recovery.

All participants received reminder texts or email messages to help adherence to standard care advice, collecting home BP measurements and IE training. The control group received monthly reminders to adhere to the standard care lifestyle advice given by their healthcare professional (HCP) at baseline. The intervention group also received monthly standard care lifestyle advice in addition to three IE training reminders per week, for the duration of the study. At follow-up time points 4 weeks, 3 months and 6 months, all participants received a 24-hour reminder to start taking their home BP.

### Data analysis

Data were analysed based on per protocol (PP) and intention-to-treat (ITT) for all patients randomised. Participants in the intervention group who completed at least two-thirds of exercise sessions at each time point were included in the PP dataset, and all participants in the control group were also included.

The primary and secondary outcomes for sBP, diastolic BP (dBP) and HR were analysed using a mixed model with repeated measures over time and an unstructured covariance matrix. This included a fixed treatment effect to compare change from baseline between IE and control groups, and adjustment was made for baseline values, sex and age (18–49, ≥50 years). This model was used to estimate differences between the treatment groups and 95% CIs. Checks for normality were carried out, and no data transformations were necessary prior to analysis.

Outcomes, demographic and baseline data were summarised to compare treatment groups. Means and SD were calculated for continuous (approximate) normally distributed variables and frequencies and percentages for categorical variables.

The correct intensity of exercise was defined as achieving a HR that fell within the 95% target. Target HR was calculated for each individual by using the equation is 95% HR _peak_+bias +/− (2.77 × CV%) where the bias was −1.5% and CV%=6.3%.^28^

The quantitative analysis was conducted using RStudio V.2023.06.1 and Stata/IC 16.1. For the economic analysis, we used the EuroQol-5 Dimensions-5 Levels (EQ-5D-5L) questionnaire. The responses were converted into utility scores using the EQ-5D-5L value set for England.^29^ Resource use was collected using the Client Service Resource Inventory. We adopted the NHS plus personal social service perspective. Unit costs of health and social care^30^ were applied to obtain individual service use data. Costs were calculated per participant using Stata 17 and Microsoft Excel (2019). The cost elements for delivering the IE intervention comprised training of clinical staff (research staff costs), and clinical staff costs to deliver the intervention and equipment (a cardboard wall squat delivery device, HR monitor and BP monitor).

## Results

This RCT met all the progression criteria outlined in the study protocol (ISRCTN: ISRCTN13472393) except target recruitment number. While recruitment was hampered by the COVID-19 pandemic, we implemented adaptations to mitigate this^26^ and recruited sufficient numbers to calculate sample size for a full trial. Only two participants were recruited through primary care.

This study showed that IE is acceptable to NHS patients/healthcare professionals as described by Rees-Roberts *et al*.^26^ There were no adverse events in the study.

Statistical summaries of the demographic data indicated that characteristics were very similar between groups. Similarly, there was no indication of differences between groups in the continuous outcomes, sBP, dBP and HR at baseline (table 1).

Screen fail rate was high (50%), 41 participants were randomised, 14 (34%) withdrew or were lost to follow-up, nine in the intervention group and five in the control group. Of note, two participants in the IE group withdrew between randomisation and prescription for reasons unrelated to the intervention.

In the PP analysis for the primary outcome, change from baseline in sBP, the difference in adjusted means between groups at 4 weeks was −3.61 mm Hg. sBP reduced in both groups compared with baseline, but a larger reduction was seen in the intervention group (−8.48 mm Hg and −4.86 mm Hg for intervention and control, respectively). Similar results were seen at 3 and 6 months; the differences between groups were −4.01 mm Hg and −2.7 mm Hg, respectively. ITT analysis supported the PP results; the between-group difference in sBP change from baseline was −2.3 mm Hg at 4 weeks, −4.07 mm Hg at 3 months and −2.3 mm Hg at 6 months. There was no change in dBP and HR (tables2  3).

**Table 2:** Estimates of treatment differences from ANCOVA (per protocol population)

	Change from baseline	Isometric exercise adj. mean (SE, n)	Control adj. mean (SE, n)	Difference (adj. mean)	Difference (95% CI)
Systolic BP(mm Hg)	4 weeks	−8.48 (3.54, 11)	−4.86 (3.01, 16)	−3.61	−13.08 to 5.86
	3 months	−8.17 (2.47, 11)	−4.15 (2.18, 14)	−4.01	−10.57 to 2.54
	6 months	−12.87 (3.78, 8)	−10.20 (3.02, 13)	−2.67	−12.65 to 7.31
Diastolic BP(mm Hg)	4 weeks	−1.07 (2.08, 11)	0.89 (1.86, 16)	−1.96	−7.60 to 3.68
	3 months	2.78 (3.91, 11)	0.88 (3.51, 14)	1.90	−8.90 to 12.70
	6 months	−1.45 (2.14, 8)	−2.28 (1.85, 13)	0.82	−5.36 to 7.00
Heart rate(bpm)	4 weeks	0.09 (2.94, 10)	−6.10 (2.43, 16)	6.19	−1.45 to 13.83
	3 months	−1.90 (3.04, 11)	−6.38 (2.75, 14)	4.49	−3.84 to 12.81
	6 months	−2.92 (3.49, 7)	−8.80 (2.83, 13)	5.88	−3.15 to 14.92

Adj, adjusted; ANCOVA, analysis of covariance; BP, blood pressure.

**Table 3:** Estimates of treatment differences from ANCOVA (intention-to-treat population)

	Change from baseline	Isometric exercise adj. mean (SE, n)	Control adj. mean (SE, n)	Difference (adj. mean)	Difference (95% CI)
Systolic BP(mm Hg)	4 weeks	−6.42 (3.22, 15)	−4.11 (3.13, 16)	−2.31	−11.29 to 6.67
	3 months	−7.58 (2.28, 15)	−3.52 (2.22, 14)	−4.07	−10.28 to 2.15
	6 months	−11.92 (3.40, 11)	−9.56 (3.13, 13)	−2.35	−11.70 to 6.99
Diastolic BP(mm Hg)	4 weeks	−0.31 (1.91, 15)	1.24 (1.90, 16)	−1.55	−6.77 to 3.66
	3 months	2.66 (3.19, 15)	1.15 (3.29, 14)	1.51	−7.73 to 10.75
	6 months	−1.23 (2.17, 11)	−1.99 (2.12, 13)	0.76	−5.40 to 6.91
Heart rate(bpm)	4 weeks	0.04 (2.52, 14)	−5.57 (2.38, 16)	5.61	−1.10 to 12.31
	3 months	−1.34 (2.66, 15)	−5.96 (2.71, 14)	4.62	−2.86 to 12.10
	6 months	−1.44 (2.87, 10)	−8.34 (2.69, 13)	6.90	−0.81 to 14.61

Adj, adjusted; ANCOVA, analysis of covariance; BP, blood pressure.

**Figure 3:**
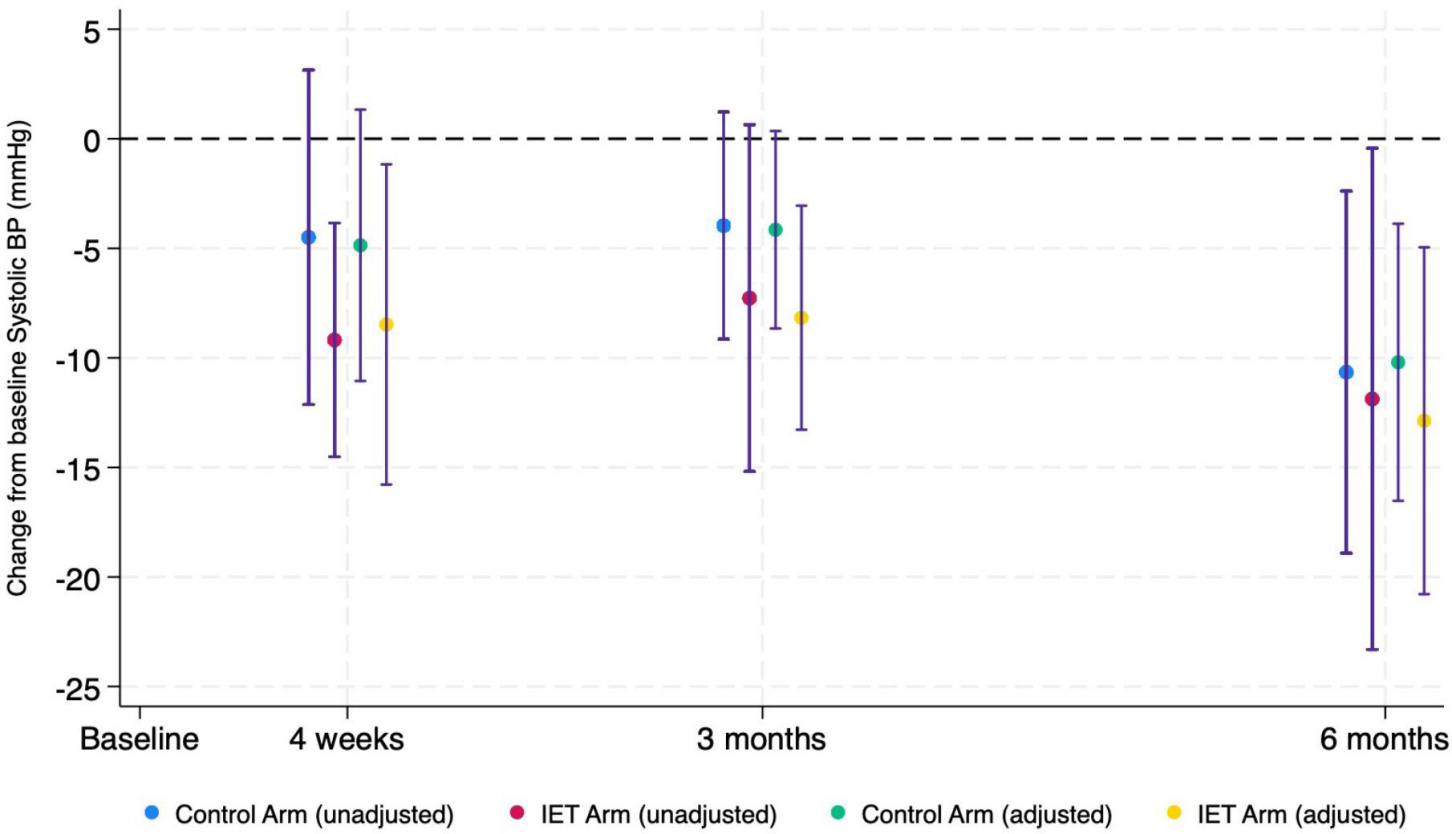
Marginal means and 95% CI from analysis of covariance (ANCOVA) (per protocol population). ANCOVA, analysis of covariance; BP, blood pressure; IET, isometric exercise test.

The study did not detect a difference between groups at any time point, because it was not powered to. However, post hoc analysis investigating the difference in sBP between baseline and each time point suggested a significant reduction in the IE group at all three time points, but only at the 6-month time point in the control group (figure 3).

There was no reduction in body weight in either group. However, of note, two control participants lost a considerable amount of weight at 6 months (8.8 kg and 11.9 kg).

All HCPs who carried out an IIET with a participant passed the competency assessment.

Patient adherence with IE was measured using the HR responses in bouts three and four of each session and was deemed adequate if at least two-thirds of HRs were in THRR. 13 of the IE participants returned HR data throughout the study. At 4 weeks, 69% of the 12 training sessions were in range; at 3 months, 73% and at 6 months, 85% of the 72 sessions.

Estimates of the SD for sBP change from baseline at 4 weeks, 3 months and 6 months are 12.5, 10.8 and 14.4 mm Hg, respectively.

### Health economic analysis

21 participants completed an EQ-5D-5L questionnaire at baseline and at least one more time point. There was no difference in QOL between groups at any time point, suggesting at this stage, based on the limited data available, that the intervention did not adversely impact QOL.

The changes in resource use between groups and over time were small. Participants did not use any hospital or social care services during the study period but did access primary care services. The estimated excess resource-use costs for the intervention at 6 months were £0.60, but this was not statistically significant at any time point.

Training and prescription delivery cost was calculated based on the average hourly wage of staff involved. For the entire study, the HCP training costs totalled £1046. The cost of delivering the intervention (performing the incremental IE test, calculating the optimal knee angle and adjusting the prescription) totalled £680.38. Equipment costs outside a research setting were calculated at £743.82 without the inclusion of the BP monitors and at £1741.52 with the inclusion of the BP monitors. The average cost for delivering the intervention was £64.73 per person without including the BP monitor cost and £110.00 per person including the BP monitor cost.

## Discussion

This is the first study to assess the feasibility of delivering an IE intervention to NHS unmedicated hypertensive patients. Recruitment through primary care was challenging, resulting in a mitigation which was to move to direct to participant recruitment (mainly through social media) and through secondary care hypertension clinics, discussed in detail in Farmer *et al*.^27^ This has led us to adapt our recruitment strategy for a full trial accordingly.

The results allow a sample size calculation for a future real-world large-scale RCT. If we accept a minimum clinically important difference in sBP of 5 mm Hg,^24^ using the 6-month SD of 14.4 mm Hg, 352 participants will attain 90% power with a 5% statistical significance level. With 35% attrition, an RCT would require 542 randomised. This finding suggests that SD values for change in BPs are above ±14 mm Hg, supporting the conclusion that some previous IE training studies may have been underpowered.^31^ This was a feasibility study and as such was not specifically powered to detect differences in BP. However, exploratory post hoc analysis comparing BP at baseline and subsequent time points showed a trend to reduced BP at all time points in the IE group (ITT=−11.9 mm Hg at 6 months), which is consistent with previous results.^23^ These sBP reductions also support previous findings that IE lowers BP equally in males and females.^32^

Few studies have investigated the effects of IE performed over longer durations on BP in hypertensive participants. Two comparable studies examined the effect of IE (handgrip) performed at 30% maximal voluntary contraction over 12 weeks^33 34^ and neither showed significant reductions compared with control. While there are numerous reasons that might explain these findings, it may be that not all IEs have the same effect on BP. A potential physiological stimulus for BP adaptation may be linked to postexercise hypotension.^35^ The wall squat has been associated with greater postexercise hypotension than isometric handgrip^36^ and results in more BP reduction following training.^24^ This is reflected in the results of another real-world study comparing the effects of 12 weeks of hand grip against wall squat IE and a control group in unmedicated hypertensive participants.^37^ The study demonstrated significant office sBP reductions of –11.2 mm Hg for hand grip and –12.9 mm Hg for wall squat groups compared with control (–4 mm Hg) but reported a larger magnitude of difference between the wall squat and control. Furthermore, the only study to investigate longitudinal anti-hypertensive efficacy of IE training beyond this duration showed significant reductions (p<0.001) in clinic sBP (−9 mm Hg) and dBP (−7 mm Hg) compared with control following 1 year of isometric wall squat training in prehypertensive males.^38^

We demonstrated low attrition at 6 months when compared with anti-hypertensive medication, which is estimated to be around 50%^39^ and even lower (around 37%) in younger adults,^40^ and the general finding that around 50% of people who start an exercise programme discontinue within 6 months.^16^ Our findings suggest that 65% of those prescribed IE continued exercising to target HR at 6 months. This compares favourably to the findings of Siada *et al*^41^ who assessed the long-term adherence of a very similar ‘at-risk’ demographic to a 12-week mixed exercise training programme and found that only 48% of participants met the adherence criteria defined as attending more than 75% of the 24 sessions. While the current study had a higher level of attrition than the 27% reported by O’Driscoll *et al*,^38^ it must be remembered that the latter was a laboratory-controlled study with direct contact between the researchers and participants as opposed to a real-world study recruiting a heterogeneous population better reflecting that of the general hypertensive population.^42^ Real-world randomised controlled exercise intervention design is extremely difficult to achieve, not least in relation to the control group, who for simplicity of comparison would ideally remain sedentary. However, for the purposes of this investigation, mandated physical inactivity would not be recognised as either standard care or an effective clinical option.^43^ Like any RCT in hypertension, a trend for reduction in BP^44^ was also seen in the control group (ITT=−9.6 mm Hg). Both groups were given detailed written instructions in relation to lifestyle advice in line with National Institute of Health and Care Excellence (NICE) 2019 and American Heart Association (AHA)^45^ physical activity guidelines for hypertension. This may have been expected based solely on the Hawthorne effect, but in this instance, it was exacerbated by the fact that people who volunteer for an exercise study will often undertake exercise if allocated to the control group. This problem of control group contamination is well documented in RCTs aiming at increasing physical activity levels^46^ and may account for a potential control group drift to taking on exercise. Indeed, the dietary advice and the general exercise recommendation that form the basis of standard care advice, if adhered to, have been consistently proven to reduce BP.^24 47^ It has been shown that using information and communication technology (aspects of which were also used in our study) to support hypertension management can significantly enhance BP control after 6 months using anti-hypertensive medication.^48^ However, it is important to acknowledge that maintaining a physically active lifestyle has many benefits, beyond lowering BP. Cautioning against the promotion of short duration exercise protocols as a substitute for a healthy lifestyle.^49^

The BP findings of this feasibility study lend further support for a large-scale RCT to inform any future changes in physical activity guidelines for the prevention and treatment of hypertension. The fact that around 40%–50% of people treated for hypertension still fail to achieve their target BP^1^ despite available clinical practice guidelines has led to the recognition that in patients with hypertension, exercise should be individually prescribed based on initial BP level.^50^ Others have reported that the incorporation of light IE training has been shown to elicit beneficial effects with very limited adverse events in those with cardiovascular disease.^51^ Indeed, there were no adverse events associated with IE in our study which supports previous findings;^52^ however, the study only included people with little or no comorbidity and uncomplicated hypertension not on treatment. Isometric activity is frequently encountered in activities of daily living along with aerobic exercise. This, along with the lower myocardial work and improved myocardial perfusion during diastole,^53^ indicates that IE may be safer than other forms of exercise training.^52 54^ Notwithstanding the relative safety of IE, Hanssen and Pescatello^55^ logically identify that other moderators such as comorbidities along with individual preferences and available resources should all be considered when prescribing exercise to this population. Therefore, IE training, if shown to be effective in a large RCT, would not be advised as a stand-alone exercise treatment for hypertension, but rather as another tool in the exercise guideline armoury to support individual prescription.

Overall, it was feasible to collect enough resource use and EQ-5D-5L data to conduct the economic analysis, despite the challenges imposed by the COVID-19 pandemic.^26^ The use of self-completed questionnaires for both resource use and health data proved a good way to collect these data. We had a 64% response rate at baseline which dropped at subsequent assessment points. We had the highest number of observations at 3 months and the lowest one at 6 months. The analysis indicated that there was no statistically significant difference in effectiveness and resource utilisation costs between the two trial groups, but the feasibility study did not have the necessary statistical power to find a significant effect. A larger study with a greater sample size is therefore desirable to understand better the size of the effect of the intervention on both outcomes and costs. As this is a feasibility study with a small sample size, these cost-effectiveness estimates are preliminary and not necessarily indicative of the cost-effectiveness of the intervention.

### Limitations

The lack of data on social deprivation and other equality, diversity and inclusion parameters limits generalisability. Additionally, volunteers for this type of activity tend to be a cohort interested in their health and therefore may not be completely representative of the wider population with hypertension.

In this study, there was a high screen failure rate (50%), which raises concerns about the generalisability of the calculation of variance in BP change. However, the results were in line with the published literature.^56^

Furthermore, it is impossible to blind exercise studies introducing the potential for bias in reporting and compliance. In this study, the investigators were blinded; however, supervising HCPs were not, which may have introduced bias. In the planned full RCT, all staff analysing results will be blinded.

Finally, the challenge of individuals in the control group independently adopting measures beyond the standard guidance introduces a confounding factor, as they may implement alternative interventions or lifestyle modifications independently, thereby blurring the distinction between groups.

### Conclusion

This feasibility study was never intended to be powered to detect a difference between groups; it has allowed us to calculate the sample size (n=542) for a full RCT of IE for people with hypertension. This trial had conservative exclusion criteria; however, we now feel that it is appropriate to include individuals on single BP agents (except beta-blockers) and those with co-morbidities (eg, myocardial infarction more than 3 months prior to enrolment) based on confirmation of good safety data. Results signal a sBP reduction in the IE group compared with baseline and indicate good acceptability and adherence rates to the treatment protocol. The cost of the intervention was just over £64 per patient which was predominantly the cost of HCP time. This suggests that the implementation of an isometric wall squat intervention in the NHS would be challenging in its current form because there is insufficient capacity to deliver this in primary care. Therefore, a planned RCT will test whether a newly developed low resource IE intervention can overcome these issues to become an effective treatment pathway to lower BP in hypertensive patients.

## Data Availability

Data are available upon reasonable request.
