# Supplementary material for: Feasibility randomised controlled trial to assess the delivery of a novel isometric exercise intervention for people diagnosed with uncomplicated stage 1 hypertension in the National Health Service: key quantitative findings
Source: BMJ Open. 2025 Jun 4;15(6):e091219. doi: 10.1136/bmjopen-2024-091219 (PMC12142027; doi:10.1136/bmjopen-2024-091219)
Supplement: Supplementary file 1 [file bmjopen-2024-091219.reviewer_comments.pdf]

PEER REVIEW HISTORY

BMJ Open publishes all reviews undertaken for accepted manuscripts. Reviewers are asked to complete a checklist review form and are provided with free text boxes to elaborate on their assessment. These free text comments are reproduced below.

ARTICLE DETAILS

Title (Provisional)

A feasibility randomised controlled trial to assess the delivery of a novel isometric exercise intervention for people diagnosed with uncomplicated Stage 1 hypertension in the National Health service: Key quantitative findings

Authors

Wiles, Jonathan Derek; Santer, Ellie; Rees-Roberts, Melanie; Borthwick, Rachel; Doulton, Timothy; Swift, Pauline A; Pellatt-Higgins, T; SAXBY, Katie; Mills, Ashley; Gousia, Katerina; MacInnes, Douglas; O'Driscoll, Jamie; West, Alan; Darby, John; Short, Vanessa; Farmer, C

VERSION 1 - REVIEW

**Reviewer**	**1**
**Name**	**Ji, Chen**
**Affiliation**	**University of Warwick Warwick Medical School, Warwick Clinical Trials Unit**
**Date**	**14-Jan-2025**
**COI**	**None**

Thank you for the opportunity to review the paper that reports the feasibilty RCT to assess the delivery of a novel isometric exercise intervention for people diagnosed with uncomplicated Stage 1 hypertension in the National Health Service.

The paper reported the clinical results of the feasibility RCT. However, it is not usually the way that a feasibility RCT paper would focus on. In the study protocol, a number of objectives are specified with criteria for progression, such as intervention delivery and accceptability, recruitment, attrition, adherence, feasibility of using observed home blood pressure readings, feedback of participants IE experience and focus group, etc. But the paper failed to report and discuss most of these results. Therefore, more details are needed to answer the progresion criteria questions so that the authors would be able to judge whether it is feasible to proceed to a definitive trial in the patients with stage 1 hypertension.

The authors should use CONSORT 2010 statement: extension to randomised pilot and feasibility trials to report this feasibility trial.

In addition, some of the results in the Abstract were not presented in the Results section, e.g. intervention intensity. For the variance in BP change, it will be helpful to report the variance and mean change in a table or figure 3, with and without adjustment.

**Reviewer**	**2**
**Name**	**Hejazi, Keyvan**
**Affiliation**	**Hakim Sabzevari University**
**Date**	**24-Jan-2025**
**COI**	**None**

As a reviewer, I believe this article demonstrates both innovation and quality that could warrant publication in the journal. The study presents a novel isometric exercise intervention specifically designed for individuals with uncomplicated Stage 1 hypertension, which is a significant area of interest given the high prevalence of hypertension and the challenges associated with its management.

The feasibility of delivering this intervention within the National Health Service (NHS) context is particularly noteworthy, as it addresses a gap in existing literature regarding the practical implementation of exercise interventions in a real-world healthcare setting. Additionally, the study's findings indicate good acceptability and adherence rates to the treatment protocol, which are critical factors for the success of any health intervention.

As a journal reviewer, I would like to point out several weaknesses in the manuscript:

1. The study was not powered to detect differences in blood pressure between groups, which limits the ability to draw definitive conclusions about the efficacy of the isometric exercise intervention. The authors acknowledge that the sample size calculation for a full randomized controlled trial (RCT) is based on the findings of this feasibility study, suggesting a need for 542 participants to achieve adequate power.

2. The study experienced a high screen fail rate of 50%, which raises concerns about the generalizability of the findings. Only 41 participants were ultimately randomized, and the high attrition rate (34% withdrew or were lost to follow-up) further complicates the interpretation of the results.

3. The article notes a lack of diversity in the participant population, which may limit the applicability of the findings to broader demographics. The authors mention that the volunteers for this type of activity tend to be health-conscious individuals, potentially skewing the results .

4. There is a significant concern regarding control group contamination, as participants in the control group may have independently adopted lifestyle changes or exercise routines, which could blur the distinction between the intervention and control groups. This issue is well-documented in RCTs aimed at increasing physical activity levels.

5. While the investigators were blinded, the supervising healthcare professionals were not, which introduces the potential for bias in reporting and compliance. This lack of blinding could affect the integrity of the study results.

6. primary and secondary outcome measures focused primarily on blood pressure changes, but the study did not adequately address other important health outcomes, such as quality of life or long-term adherence to the intervention. Although the EQ-5D-5L questionnaire was used, there were no significant differences in quality of life reported between groups.

**Reviewer**	**3**
**Name**	**Ferreira, Maycon**
**Affiliation**	**Universidade Federal de São Paulo**
**Date**	**28-Jan-2025**
**COI**	**None**

In the present study, the feasibility of delivering personalised isometric exercise for people with stage 1 hypertension was investigated. Based on the findings, the authors conclude that "results signal a sBP reduction in the IE group compared to baseline and indicate good acceptability and adherence rates to the treatment protocol."

Comments:

- The monitoring of blood pressure (BP) via video is a topic that requires further clarification in the current version of the article. I suggest the authors elaborate in more detail on the process and potential biases.

- An important point the authors should consider in the discussion is the practice of exercise as a lifestyle habit, rather than merely a means of reducing blood pressure. It is necessary to emphasize that maintaining a physically active lifestyle contributes to countless systemic benefits, including hemodynamic ones (such as lowering blood pressure). Short protocols using isometric exercise have been suggested as effective for blood pressure control. However, greater care is needed with the message conveyed through "quick" physical training protocols with isometric exercises, as they may erroneously promote the idea that small amounts of exercise are sufficient for an individual’s overall quality of life, as recently highlighted (10.1093/ajh/hpae036).

VERSION 1 - AUTHOR RESPONSE

Reviewer: 1
Dr. Chen Ji, University of Warwick Warwick Medical School
Comments to the Author:
Thank you for the opportunity to review the paper that reports the feasibilty RCT to assess the delivery of a novel isometric exercise intervention for people diagnosed with uncomplicated Stage 1 hypertension in the National Health Service.

The paper reported the clinical results of the feasibility RCT. However, it is not usually the way that a feasibility RCT paper would focus on. In the study protocol, a number of objectives are specified with criteria for progression, such as intervention delivery and accceptability, recruitment, attrition, adherence, feasibility of using observed home blood pressure readings, feedback of participants IE experience and focus group, etc. But the paper failed to report and discuss most of these results. Therefore, more details are needed to answer the progresion criteria questions so that the authors would be able to judge whether it is feasible to proceed to a definitive trial in the patients with stage 1 hypertension.

The authors should use CONSORT 2010 statement: extension to randomised pilot and feasibility trials to report this feasibility trial.

In addition, some of the results in the Abstract were not presented in the Results section, e.g. intervention intensity. For the variance in BP change, it will be helpful to report the variance and mean change in a table or figure 3, with and without adjustment.

We thank the reviewer for their helpful comments. We agree that the title of this paper is too broad and implies it covers all results of the pilot study, this was not possible in a single paper due to word count limitations. We have altered the tile accordingly by adding “key quantitative findings” to reflect this and have added a text in the discussion to highlight the fact that two other papers cover the qualitative feasibility aspects and a discussion relating to recruitment difficulties in COVID when undertaking this study. The two additional papers are:

Rees-Roberts, M., Borthwick, R., Santer, E. et al. Experiences, acceptability and feasibility of an isometric exercise intervention for stage 1 hypertension: embedded qualitative study in a randomised controlled feasibility trial. Pilot Feasibility Stud 10, 113 (2024). https://doi.org/10.1186/s40814-024-01539-8

Farmer C, Santer E, West A, et al. Delivering clinical studies of exercise in the COVID-19 pandemic: challenges and adaptations using a feasibility trial of isometric exercise to treat hypertension as an exemplar. BMJ Open 2023;13:e068204. doi:10.1136/ bmjopen-2022-068204

Regarding the intervention intensity discussed in the abstract, the results were included in the original submission in the main body text. We have added an explanation of calculation (with reference) to the methods section of the paper.

We also note a typo in the abstract completion should read 85% not 87%.

We have updated the abstract to be in line with the CONSORT extension to randomised pilot and feasibility trials guidance document. However, the subtitles in the abstract follow BMJ guidance rather than those outlined in consort, e.g., “design” as opposed to “trial design”.

We have updated the introduction to cover the main aims of the paper in relation to progression to full RCT.

Our original protocol paper (doi.org/10.1186/s40814-021) included a list of primary and secondary endpoints. It is not possible to cover all aspects of this trial in a single paper mainly

due of word limitations. We have covered recruitment difficulties and adaptions in relation to the COVID-19 pandemic in Farmer et al. (doi:10.1136/ bmjopen-2022-068204) and the qualitative results in Rees-Roberts et al. (https://doi.org/10.1186/s40814-024-01539-8).

The primary objectives in the protocol paper were as follows:

1. To assess if healthcare professionals (e.g. nurses, health trainer, healthcare assistants, physiotherapists) can deliver isometric exercise prescriptions for stage 1 hypertensive patients in a primary care NHS setting

This is covered in the paper listed above by Rees-Roberts et al. This objective was erroneously included in our quantitative paper and we have removed it from the text.

2. To estimate the variance in BP change, to enable sample size calculation for a definitive randomised controlled trial

This outcome is included in the paper we have included variance in BP change and mean change in a table or figure 3, with and without adjustment.

The secondary objectives are as follows:

1. Evidence the fidelity of the study intervention with respect to healthcare professional delivery and patient completion of IE

This is discussed in Rees-Roberts et al.

2. Estimate short- (4 week) and medium-term (3 and 6 month) adherence rates to IE intervention

We have included adherence rates in paragraph 8 of the results section.

This is discussed in this paper.

3. Estimate recruitment and attrition rates at recruiting GP sites to inform future trials

Recruitment through GP sites was very limited in part due to the COVID pandemic. This is covered in doi:10.1136/ bmjopen-2022-068204 in some detail. As a result our full study is using direct to participant recruitment strategies which is also discussed in the aforementioned paper. We have now added a section in the results and discussion to cover this.

4. Explore the willingness of GPs, secondary care clinicians and healthcare professionals to consider IE as a treatment option for patients, including barriers and facilitators for delivering and integrating this within an NHS care pathway for hypertension

This is covered in the paper by Rees-Roberts et al.

5. Establish the cost and cost-utility of the IE intervention compared to standard care for stage 1 hypertension

This is included in the paper.

6. Understand participant experiences of undertaking IE, adherence to the programme and continuation. Additionally, explore possible negative effects of COVID-19 on recruitment rates and participation

This is covered in the paper by Rees-Roberts et al.

7. To investigate the feasibility of using observed home blood pressure readings for remote monitoring

This is covered in the paper by Rees-Roberts et al.

We would suggest that the combination of the three papers (two previously published and this one submitted) discussed cover the main guidelines raised in Eldridge et al. (BMJ 2016; 355 doi: https://doi.org/10.1136/bmj.i5239) along with the changes to this submitted paper as discussed in the response.

Reviewer: 2
Dr. Keyvan Hejazi, Hakim Sabzevari University
Comments to the Author:

As a reviewer, I believe this article demonstrates both innovation and quality that could warrant publication in the journal. The study presents a novel isometric exercise intervention specifically designed for individuals with uncomplicated Stage 1 hypertension, which is a significant area of interest given the high prevalence of hypertension and the challenges associated with its management.
The feasibility of delivering this intervention within the National Health Service (NHS) context is particularly noteworthy, as it addresses a gap in existing literature regarding the practical implementation of exercise interventions in a real-world healthcare setting. Additionally, the study's findings indicate good acceptability and adherence rates to the treatment protocol, which are critical factors for the success of any health intervention.

As a journal reviewer, I would like to point out several weaknesses in the manuscript:

1. The study was not powered to detect differences in blood pressure between groups, which limits the ability to draw definitive conclusions about the efficacy of the isometric exercise intervention. The authors acknowledge that the sample size calculation for a full randomized controlled trial (RCT) is based on the findings of this feasibility study, suggesting a need for 542 participants to achieve adequate power.

We thank the author for their helpful comments. We agree that the study was not powered to detect differences between the two groups, the primary aim was to allow us to calculate sample size for a full RCT. We have highlighted that the study was not powered in the abstract, objectives, results and discussion. We have included a further comment in the conclusion highlighting the initial trial design.

2. The study experienced a high screen fail rate of 50%, which raises concerns about the generalizability of the findings. Only 41 participants were ultimately randomized, and the high attrition rate (34% withdrew or were lost to follow-up) further complicates the interpretation of the results.

We agree there was a high screen failure rate, this was primarily due to conservative inclusion and exclusion criteria. This result has allowed us to modify our protocol for the full study of 542 people. The primary aim of the study was to determine the variance in BP change, to enable a sample size calculation for a randomised controlled trial (RCT). There is a danger that the screen failure rate may have skewed this estimation, we have acknowledged this in the paper in the limitation section.

3. The article notes a lack of diversity in the participant population, which may limit the applicability of the findings to broader demographics. The authors mention that the volunteers for this type of activity tend to be health-conscious individuals, potentially skewing the results.

Thank you for highlighting this inherent issue, we acknowledge this comment and have highlighted this in the limitation section in the paper. This is a potential issue with any exercise intervention and it is entirely possible that when the full RCT is completed the intervention will only be applicable to those who will do it. IE if shown effective would be an intervention to add to existing treatments pathways and may not be appropriate for all eligible people with hypertension.

4. There is a significant concern regarding control group contamination, as participants in the control group may have independently adopted lifestyle changes or exercise routines, which could blur the distinction between the intervention and control groups. This issue is well-documented in RCTs aimed at increasing physical activity levels.

We agree and this has been acknowledged in the paper in the limitations section.

5. While the investigators were blinded, the supervising healthcare professionals were not, which introduces the potential for bias in reporting and compliance. This lack of blinding could affect the integrity of the study results.

We agree and this has been acknowledged in the paper in the limitations section.

6. primary and secondary outcome measures focused primarily on blood pressure changes, but the study did not adequately address other important health outcomes, such as quality of life or long-term adherence to the intervention. Although the EQ-5D-5L questionnaire was used, there were no significant differences in quality of life reported between groups.

Quality of life was included in this feasibility study to ensure that there was no signal that IE had a deleterious effect on QOL. We agree this is an important consideration and have now commented on this in the health economic section. We agree that exploring long term adherence is important and this has been included in the full RCT.

Reviewer: 3
Dr. Maycon Ferreira, Universidade Federal de São Paulo
Comments to the Author:
In the present study, the feasibility of delivering personalised isometric exercise for people with stage 1 hypertension was investigated. Based on the findings, the authors conclude that "results signal a sBP reduction in the IE group compared to baseline and indicate good acceptability and adherence rates to the treatment protocol."

Comments:

- The monitoring of blood pressure (BP) via video is a topic that requires further clarification in the current version of the article. I suggest the authors elaborate in more detail on the process and potential biases.

Thank you for your helpful comment, as requested we have included more detail in the intervention section.

- An important point the authors should consider in the discussion is the practice of exercise as a lifestyle habit, rather than merely a means of reducing blood pressure. It is necessary to emphasize that maintaining a physically active lifestyle contributes to countless systemic benefits, including hemodynamic ones (such as lowering blood pressure). Short protocols using isometric exercise have been suggested as effective for blood pressure control. However, greater care is needed with the message conveyed through "quick" physical training protocols with isometric exercises, as they may erroneously promote the idea that small amounts of exercise are sufficient for an individual’s overall quality of life, as recently highlighted

(10.1093/ajh/hpae036).

Thank you for providing this helpful comment and reference. We have now included this caveat in the discussion section which we believe strengthens it. We have now referenced American Journal of Hypertension, Volume 37, Issue 6, June 2024, Pages 394–395 [Reference 48 in the text]

VERSION 2 - REVIEW

**Reviewer**	**1**
**Name**	**Ji, Chen**
**Affiliation**	**University of Warwick Warwick Medical School, Warwick Clinical Trials Unit**
**Date**	**02-Apr-2025**
**COI**	

Thanks for the updated manuscript and responses to the previous comments. The authors have made significant efforts to clarify the objectives and incorporate relevant results, which greatly aid the interpretation of trial feasibility. These improvements are highly valuable for reviewing this manuscript.

I have a few minor comments below:

1. CONSORT Checklist: The authors have updated the abstract to align with the CONSORT guidelines for feasibility trials. Could the authors also adapt the attached checklist to the version for pilot trials? This would help highlight additional relevant information, such as details on “how participants were identified and consented” and the “implications for progression from pilot to future definitive trials, including any proposed amendments.”

2. Abstract Line 8: Should “Is feasible to deliver an isometric wall squat …” be revised to “Is it feasible to deliver an isometric wall squat …”?

3. Abstract Line 28: The mean age should be corrected to 57.

4. Page 4 Lines 11-14: Thank you for clarifying the objective of this paper here. It may be useful to mention that some of the qualitative results have already been published (Rees-Roberts et al. & Farmer et al.). I see this has been added just before the limitations. Maybe just move the text here.

5. Page 6: The summary of patient characteristics could be moved to the Results section. While the exclusion criteria are summarised, it would also be useful to present the inclusion criteria in the main text. Additionally, please clarify the settings and locations where data were collected (e.g., how data were collected for patients in the control arm) and how participants were identified and consented (as per the CONSORT checklist for pilot trials).

6. Page 8 Line 11: It may be preferable to mention the per-protocol analysis first to match the presentation order of the results.

7. Page 8 Lines 12-13: Is this the definition of intervention adherence? Some clarification may be needed:

o It is unclear whether the “two-thirds of the exercise sessions” rule applies only between baseline and the first four weeks.

o How were patients excluded from the per protocol analysis based on this rule? Does it apply to anyone who failed to complete two-thirds of sessions in any four-week period, or only to those who completed <48 out of 72 sessions over the entire six-month follow-up?

8. Page 8 Line 20: The authors state that outcomes were analysed with adjustments for baseline values, sex, and age. However, in Tables 2.1 and 2.2, it appears that differences from baseline were modelled. If so, this represents a change from the protocol, and the resulting estimates may differ (even if the difference is small).

9. Page 8 Line 43: What is a “carboard” wall squat delivery device? Is this a typo?

10. Page 8 Line 50: The authors state that the trial met all progression criteria. Could a reference be provided if this evidence has been published elsewhere?

11. Page 8 Lines 54-55: Could the authors provide a reference for the statement that “this study showed that IE is acceptable to NHS patients/healthcare professionals”? Does this refer to the Rees-Roberts paper?

12. Page 8 Line 60: Weight and BMI were not listed as outcomes in the protocol. Some clarification may be needed here.

13. Page 9 Line 15: “Table 2” should be corrected to “Tables 2.1 and 2.2.” or just use Table 2 and Table 3.

14. Page 9 Table 2.1: It would be helpful to include the number of patients in each arm and each analysis. Could the authors also add the standard deviations for the adjusted means in both the intervention and control arms?

15. Page 10 Table 2.2: Similar to Table 2.1, please include the number of patients in each arm and analysis, as well as the standard deviations for the adjusted means.

16. Page 10 Line 44: Do the authors mean a “significant” reduction? Both treatments showed some reduction across the three follow-ups.

17. Page 10 Lines 52-53: Consider replacing “compliance” with “adherence” for consistency.

18. Recruitment rate: It is unclear whether the recruitment rate was discussed in Farmer et al. or Rees-Roberts et al. If not, could the authors address this? As recruitment rate is one of the study objectives, discussing it could provide valuable insights for designing the next stage of the trial.

VERSION 2 - AUTHOR RESPONSE

Reviewer: 1
Dr. Chen Ji, University of Warwick Warwick Medical School
Comments to the Author:
Thank you for the opportunity to review the paper that reports the feasibilty RCT to assess the delivery of a novel isometric exercise intervention for people diagnosed with uncomplicated Stage 1 hypertension in the National Health Service.

The paper reported the clinical results of the feasibility RCT. However, it is not usually the way that a feasibility RCT paper would focus on. In the study protocol, a number of objectives are specified with criteria for progression, such as intervention delivery and accceptability, recruitment, attrition, adherence, feasibility of using observed home blood pressure readings, feedback of participants IE experience and focus group, etc. But the paper failed to report and discuss most of these results. Therefore, more details are needed to answer the progresion criteria questions so that the authors would be able to judge whether it is feasible to proceed to a definitive trial in the patients with stage 1 hypertension.

The authors should use CONSORT 2010 statement: extension to randomised pilot and feasibility trials to report this feasibility trial.

In addition, some of the results in the Abstract were not presented in the Results section, e.g. intervention intensity. For the variance in BP change, it will be helpful to report the variance and mean change in a table or figure 3, with and without adjustment.

We thank the reviewer for their helpful comments. We agree that the title of this paper is too broad and implies it covers all results of the pilot study, this was not possible in a single paper due to word count limitations. We have altered the tile accordingly by adding “key quantitative findings” to reflect this and have added a text in the discussion to highlight the fact that two other papers cover the qualitative feasibility aspects and a discussion relating to recruitment difficulties in COVID when undertaking this study. The two additional papers are:

Rees-Roberts, M., Borthwick, R., Santer, E. et al. Experiences, acceptability and feasibility of an isometric exercise intervention for stage 1 hypertension: embedded qualitative study in a randomised controlled feasibility trial. Pilot Feasibility Stud 10, 113 (2024). https://doi.org/10.1186/s40814-024-01539-8

Farmer C, Santer E, West A, et al. Delivering clinical studies of exercise in the COVID-19 pandemic: challenges and adaptations using a feasibility trial of isometric exercise to treat hypertension as an exemplar. BMJ Open 2023;13:e068204. doi:10.1136/ bmjopen-2022-068204

Regarding the intervention intensity discussed in the abstract, the results were included in the original submission in the main body text. We have added an explanation of calculation (with reference) to the methods section of the paper.

We also note a typo in the abstract completion should read 85% not 87%.

We have updated the abstract to be in line with the CONSORT extension to randomised pilot and feasibility trials guidance document. However, the subtitles in the abstract follow BMJ guidance rather than those outlined in consort, e.g., “design” as opposed to “trial design”.

We have updated the introduction to cover the main aims of the paper in relation to progression to full RCT.

Our original protocol paper (doi.org/10.1186/s40814-021) included a list of primary and secondary endpoints. It is not possible to cover all aspects of this trial in a single paper mainly due of word limitations. We have covered recruitment difficulties and adaptions in relation to the COVID-19 pandemic in Farmer et al. (doi:10.1136/ bmjopen-2022-068204) and the qualitative results in Rees-Roberts et al. (https://doi.org/10.1186/s40814-024-01539-8).

The primary objectives in the protocol paper were as follows:

3. To assess if healthcare professionals (e.g. nurses, health trainer, healthcare assistants, physiotherapists) can deliver isometric exercise prescriptions for stage 1 hypertensive patients in a primary care NHS setting

This is covered in the paper listed above by Rees-Roberts et al. This objective was erroneously included in our quantitative paper and we have removed it from the text.

4. To estimate the variance in BP change, to enable sample size calculation for a definitive randomised controlled trial

This outcome is included in the paper we have included variance in BP change and mean change in a table or figure 3, with and without adjustment.

The secondary objectives are as follows:

8. Evidence the fidelity of the study intervention with respect to healthcare professional delivery and patient completion of IE

This is discussed in Rees-Roberts et al.

9. Estimate short- (4 week) and medium-term (3 and 6 month) adherence rates to IE intervention

We have included adherence rates in paragraph 8 of the results section.

This is discussed in this paper.

10. Estimate recruitment and attrition rates at recruiting GP sites to inform future trials

Recruitment through GP sites was very limited in part due to the COVID pandemic. This is covered in doi:10.1136/ bmjopen-2022-068204 in some detail. As a result our full study is using direct to participant recruitment strategies which is also discussed in the aforementioned paper. We have now added a section in the results and discussion to cover this.

11. Explore the willingness of GPs, secondary care clinicians and healthcare professionals to consider IE as a treatment option for patients, including barriers and facilitators for delivering and integrating this within an NHS care pathway for hypertension

This is covered in the paper by Rees-Roberts et al.

12. Establish the cost and cost-utility of the IE intervention compared to standard care for stage 1 hypertension

This is included in the paper.

13. Understand participant experiences of undertaking IE, adherence to the programme and continuation. Additionally, explore possible negative effects of COVID-19 on recruitment rates and participation

This is covered in the paper by Rees-Roberts et al.

14. To investigate the feasibility of using observed home blood pressure readings for remote monitoring

This is covered in the paper by Rees-Roberts et al.

We would suggest that the combination of the three papers (two previously published and this one submitted) discussed cover the main guidelines raised in Eldridge et al. (BMJ 2016; 355 doi: https://doi.org/10.1136/bmj.i5239) along with the changes to this submitted paper as discussed in the response.

Reviewer: 2
Dr. Keyvan Hejazi, Hakim Sabzevari University
Comments to the Author:

As a reviewer, I believe this article demonstrates both innovation and quality that could warrant publication in the journal. The study presents a novel isometric exercise intervention specifically designed for individuals with uncomplicated Stage 1 hypertension, which is a significant area of interest given the high prevalence of hypertension and the challenges associated with its management.
The feasibility of delivering this intervention within the National Health Service (NHS) context is particularly noteworthy, as it addresses a gap in existing literature regarding the practical implementation of exercise interventions in a real-world healthcare setting. Additionally, the study's findings indicate good acceptability and adherence rates to the treatment protocol, which are critical factors for the success of any health intervention.

As a journal reviewer, I would like to point out several weaknesses in the manuscript:

1. The study was not powered to detect differences in blood pressure between groups, which limits the ability to draw definitive conclusions about the efficacy of the isometric exercise intervention. The authors acknowledge that the sample size calculation for a full randomized controlled trial (RCT) is based on the findings of this feasibility study, suggesting a need for 542 participants to achieve adequate power.

We thank the author for their helpful comments. We agree that the study was not powered to detect differences between the two groups, the primary aim was to allow us to calculate sample size for a full RCT. We have highlighted that the study was not powered in the abstract, objectives, results and discussion. We have included a further comment in the conclusion highlighting the initial trial design.

2. The study experienced a high screen fail rate of 50%, which raises concerns about the generalizability of the findings. Only 41 participants were ultimately randomized, and the high attrition rate (34% withdrew or were lost to follow-up) further complicates the interpretation of the results.

We agree there was a high screen failure rate, this was primarily due to conservative inclusion and exclusion criteria. This result has allowed us to modify our protocol for the full study of 542 people. The primary aim of the study was to determine the variance in BP change, to enable a sample size calculation for a randomised controlled trial (RCT). There is a danger that the screen failure rate may have skewed this estimation, we have acknowledged this in the paper in the limitation section.

3. The article notes a lack of diversity in the participant population, which may limit the

applicability of the findings to broader demographics. The authors mention that the volunteers for this type of activity tend to be health-conscious individuals, potentially skewing the results.

Thank you for highlighting this inherent issue, we acknowledge this comment and have highlighted this in the limitation section in the paper. This is a potential issue with any exercise intervention and it is entirely possible that when the full RCT is completed the intervention will only be applicable to those who will do it. IE if shown effective would be an intervention to add to existing treatments pathways and may not be appropriate for all eligible people with hypertension.

4. There is a significant concern regarding control group contamination, as participants in the control group may have independently adopted lifestyle changes or exercise routines, which could blur the distinction between the intervention and control groups. This issue is well-documented in RCTs aimed at increasing physical activity levels.

We agree and this has been acknowledged in the paper in the limitations section.

5. While the investigators were blinded, the supervising healthcare professionals were not, which introduces the potential for bias in reporting and compliance. This lack of blinding could affect the integrity of the study results.

We agree and this has been acknowledged in the paper in the limitations section.

6. primary and secondary outcome measures focused primarily on blood pressure changes, but the study did not adequately address other important health outcomes, such as quality of life or long-term adherence to the intervention. Although the EQ-5D-5L questionnaire was used, there were no significant differences in quality of life reported between groups.

Quality of life was included in this feasibility study to ensure that there was no signal that IE had a deleterious effect on QOL. We agree this is an important consideration and have now commented on this in the health economic section. We agree that exploring long term adherence is important and this has been included in the full RCT.

Reviewer: 3
Dr. Maycon Ferreira, Universidade Federal de São Paulo
Comments to the Author:
In the present study, the feasibility of delivering personalised isometric exercise for people with stage 1 hypertension was investigated. Based on the findings, the authors conclude that "results signal a sBP reduction in the IE group compared to baseline and indicate good acceptability and adherence rates to the treatment protocol."

Comments:

- The monitoring of blood pressure (BP) via video is a topic that requires further clarification in the current version of the article. I suggest the authors elaborate in more detail on the process and potential biases.

Thank you for your helpful comment, as requested we have included more detail in the intervention section.

- An important point the authors should consider in the discussion is the practice of exercise as a lifestyle habit, rather than merely a means of reducing blood pressure. It is necessary to emphasize that maintaining a physically active lifestyle contributes to countless systemic benefits, including hemodynamic ones (such as lowering blood pressure). Short protocols using isometric exercise have been suggested as effective for blood pressure control. However, greater care is needed with the message conveyed through "quick" physical training protocols with isometric exercises, as they may erroneously promote the idea that small amounts of exercise

are sufficient for an individual's overall quality of life, as recently highlighted (10.1093/ajh/hpae036).

Thank you for providing this helpful comment and reference. We have now included this caveat in the discussion section which we believe strengthens it. We have now referenced American Journal of Hypertension, Volume 37, Issue 6, June 2024, Pages 394–395 [Reference 48 in the text]
